# The functional landscape of mouse gene expression

**DOI:** 10.1186/jbiol16

**Published:** 2004-12-06

**Authors:** Wen Zhang, Quaid D Morris, Richard Chang, Ofer Shai, Malina A Bakowski, Nicholas Mitsakakis, Naveed Mohammad, Mark D Robinson, Ralph Zirngibl, Eszter Somogyi, Nancy Laurin, Eftekhar Eftekharpour, Eric Sat, Jörg Grigull, Qun Pan, Wen-Tao Peng, Nevan Krogan, Jack Greenblatt, Michael Fehlings, Derek van der Kooy, Jane Aubin, Benoit G Bruneau, Janet Rossant, Benjamin J Blencowe, Brendan J Frey, Timothy R Hughes

**Affiliations:** 1 Banting and Best Department of Medical Research, University of Toronto, 1 King's College Circle, Toronto, ON M5S 1A8, Canada; 2 Department of Medical Genetics and Microbiology, University of Toronto, 1 King's College Circle, Toronto, ON M5S 1A8, Canada; 3 Department of Electrical and Computer Engineering, University of Toronto, 1 King's College Circle, Toronto, ON M5S 1A8, Canada; 4 Department of Surgery, University of Toronto, 1 King's College Circle, Toronto, ON M5S 1A8, Canada; 5 Samuel Lunenfeld Research Institute, Mount Sinai Hospital, 600 University Avenue, Toronto, ON M5G 1X5, Canada; 6 Division of Cell and Molecular Biology, Toronto Western Research Institute and Krembil Neuroscience Center, 399 Bathurst St., Toronto, ON M5T 2S8, Canada; 7 The Hospital for Sick Children, 555 University Ave., Toronto, ON M5G 1X8, Canada

## Abstract

**Background:**

Large-scale quantitative analysis of transcriptional co-expression has been used to dissect regulatory networks and to predict the functions of new genes discovered by genome sequencing in model organisms such as yeast. Although the idea that tissue-specific expression is indicative of gene function in mammals is widely accepted, it has not been objectively tested nor compared with the related but distinct strategy of correlating gene co-expression as a means to predict gene function.

**Results:**

We generated microarray expression data for nearly 40,000 known and predicted mRNAs in 55 mouse tissues, using custom-built oligonucleotide arrays. We show that quantitative transcriptional co-expression is a powerful predictor of gene function. Hundreds of functional categories, as defined by Gene Ontology 'Biological Processes', are associated with characteristic expression patterns across all tissues, including categories that bear no overt relationship to the tissue of origin. In contrast, simple tissue-specific restriction of expression is a poor predictor of which genes are in which functional categories. As an example, the highly conserved mouse gene *PWP1 *is widely expressed across different tissues but is co-expressed with many RNA-processing genes; we show that the uncharacterized yeast homolog of *PWP1 *is required for rRNA biogenesis.

**Conclusions:**

We conclude that 'functional genomics' strategies based on quantitative transcriptional co-expression will be as fruitful in mammals as they have been in simpler organisms, and that transcriptional control of mammalian physiology is more modular than is generally appreciated. Our data and analyses provide a public resource for mammalian functional genomics.

## Background

Tissue-specific gene expression has traditionally been viewed as a predictor of tissue-specific function: for example, genes specifically expressed in the eye are likely to be involved in vision. But microarray analysis in model organisms such as yeast and *Caenorhabditis elegans *has established that coordinate transcriptional regulation of functionally related genes occurs on a broader scale than was previously recognized, encompassing at least half of all cellular processes in yeast [1-5]. Consequently, gene expression patterns can be used to predict gene functions, thereby providing a starting point for the directed and systematic experimental characterization of novel genes [1-10]. As an example, it was observed in yeast that a group of more than 200 genes involved primarily in RNA processing and ribosome biogenesis is transcriptionally co-regulated, in addition to being constitutively expressed at some level [11]. Application of statistical inference methods led to the prediction that the uncharacterized genes in this co-regulated group were likely to be involved in RNA processing and/or ribosome biogenesis [5,9]. Subsequent experimental analysis using yeast mutants validated that many of these predictions were in fact accurate [9].

To date, this approach has only been extensively applied to relatively simple model organisms such as yeast and *C. elegans*. Its general utility in mammals has not yet been established with respect to the proportion of either genes or functional categories to which it can be effectively applied. Nor has it been formally examined how the use of quantitative transcriptional co-expression for inference of gene function compares to the more traditional approach of inferring functions on the basis of tissue-specific transcription. The extent and precision of hypotheses regarding gene functions that can be drawn from expression analysis in mammals is an important and timely question, given the current absence of knowledge of the physiological functions of at least half of all mammalian genes. Given that distinct and coordinate expression of a group of functionally related genes implies an underlying pathway-specific transcriptional regulatory mechanism, identification of such instances would also represent a step towards delineating mammalian transcriptional networks.

Here, in order to demarcate the general utility of using gene expression patterns to infer mammalian gene functions, and to use this information to begin characterizing genes discovered by sequencing the mouse genome [12], we used custom-built DNA oligonucleotide microarrays to generate an expression data set for nearly 40,000 known and predicted mouse mRNAs across 55 diverse tissues. Several criteria show that these data are reliable and consistent with other information about gene expression and tissue function. Cross-validation results from machine-learning algorithms show that patterns of gene co-expression within many functional categories are 'learnable' and distinct from patterns of other categories, thus proving that many functional categories are transcriptionally co-expressed and likely to be co-regulated. In contrast, tissue-specificity alone is a comparatively poor predictor of gene function, illustrating the importance of quantitative gene expression measurements. To exemplify this, we functionally characterized the highly conserved gene *PWP1*, which is widely expressed. *PWP1 *is co-expressed with many RNA-processing genes in mouse, and we show that its yeast homolog is required for rRNA biogenesis. The data and the associated analyses in this paper will be invaluable for directing experimental characterization of gene functions in mammals, as well as for dissecting the mammalian transcriptional regulatory hierarchy.

## Results

### Expression analysis of mouse XM gene sequences

In order to generate an extensive survey of mammalian gene expression, we analyzed mRNA abundance in 55 mouse tissues using custom-designed microarrays of 60-mer oligonucleotides [13] corresponding to 41,699 known and predicted mRNAs identified in the draft mouse genome sequence using gene-finding programs [12,14] (NCBI 'XM' sequences; approximately 39,309 are unique; for further details, see the Materials and methods section). Tissue collection was a collaborative effort among several labs in the Toronto area, each with expertise in distinct areas of physiology; consequently, the mouse tissues we analyzed were obtained from several different strains of mice which are typically used to study specific organs and cell types of interest (additional cell lines and fractionated cells from animals were also analyzed, but the results are not included here because the data appear to bear little relationship to the tissue of origin of the cells examined). Since it has previously been established that there is a high correlation in expression of orthologous genes between mice and humans [15], large variations in tissue-specific expression should not occur between individuals within the same species, although we cannot rule out subtle strain-specific differences. To maximize the fidelity of measurements, unamplified cDNA from at least 1 μg of polyA-purified mRNA was hybridized to each array, with fluor-reversed duplicates performed in each case. For most organs this required pooling RNA from multiple animals; for example, more than 50 mice were required to obtain sufficient prostate mRNA. Consequently, potential variations due to parameters such as circadian rhythms or individual dissections should have been minimized by averaging over multiple animals.

All hybridizations were performed in duplicate. Data processing and normalization are described in detail in the Materials and methods. The data were processed so that each measurement reflects the abundance of each transcript in each tissue relative to the median expression across all 55 tissues; although the microarray spot intensities were used to determine which genes were detected as expressed (see below), the figures herein show the normalized, arcsinh-transformed and median-subtracted data, which for convenience we refer to as ratios. All of the data, together with tables detailing correspondence to genes in other cDNA and EST databases, annotations and other features of the encoded proteins, probe sequences, and other files used in our analyses below, are available as Additional data files and without restriction on our website [16].

### Validation of expression data

**Figure 1:**
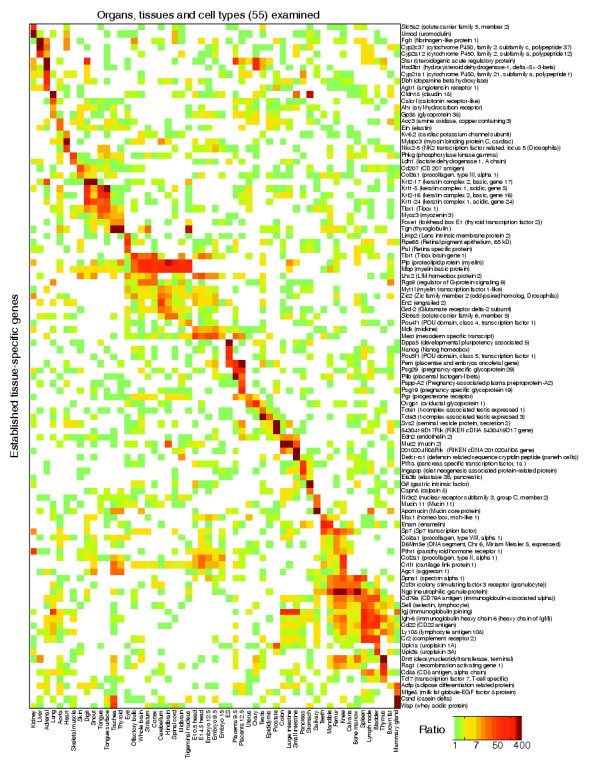
Expression of previously characterized tissue-specific genes. Genes were identified manually by searching MEDLINE abstracts [66] and XM sequence description fields (see Additional data file 1) for keywords corresponding to the appropriate tissues. Rows and columns were ordered manually.

Four lines of evidence support the quality of our data and its consistency with existing knowledge of mammalian physiology and gene expression. First, we detected the expected patterns of expression for genes previously shown to be expressed specifically in each of the 55 tissues surveyed (Figure 1). This validates the accuracy of our dissections, and indicates that there was little cross-contamination between tissue samples.

**Figure 2:**
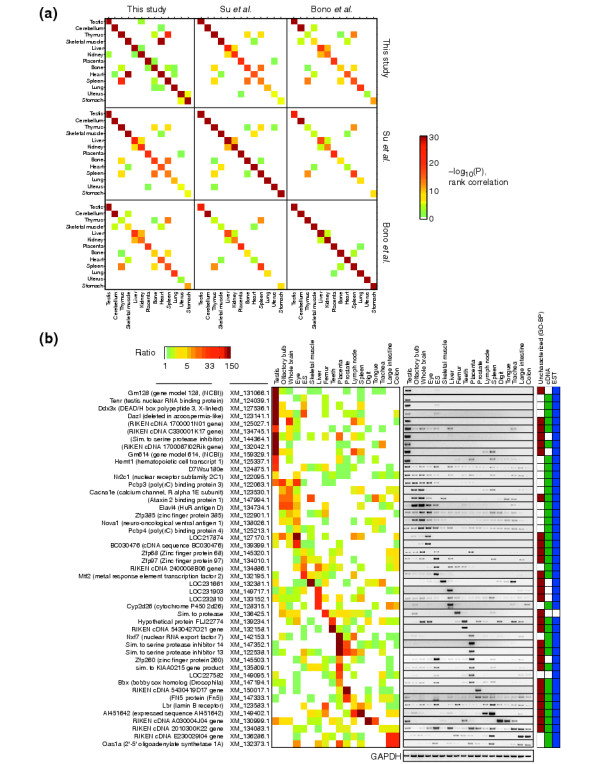
Validation of expression data by independent confirmation. **(a) **The *P *value of Spearman's Rank correlations (see Materials and methods) is shown for all possible comparisons among the 13 tissues common to all three studies (ours and those by Su *et al. *[15] and Bono *et al. *[17]) and 1,109 genes for which the same isoform is unambiguously represented on the arrays used in each of the studies (see Materials and methods). **(b) **Microarray data and RT-PCR results for 47 known and predicted XM genes are shown. Genes were selected to represent primarily those without GO Biological Processes (GO-BP) assignment and to encompass expression in all 18 tissues, and were biased towards those with functions predicted by support vector machines (SVMs) in categories of interest (or expressed in tissues of interest). The three columns on the far right show whether each XM gene was uncharacterized (not annotated) in GO-BP, and whether it is represented by a cDNA or EST.

Second, there is a clear correspondence, albeit not absolute, between our data and two other mouse microarray data sets [15,17], which surveyed a subset of the genes and tissues that we have examined. Thirteen tissues and 1,109 genes were unambiguously shared among the three studies (Figure 2a). Our data are more highly correlated with those of Su *et al. *[15], who also employed oligonucleotide array technology, whereas Bono *et al. *[17] used spotted cDNAs (Figure 2a). Furthermore, our data are more highly correlated with either of the two other studies than the two other studies are to one another. (It should be noted that these previous studies did not examine the use of transcriptional co-expression to predict gene function, which is the focus of the present study.)

Third, our array data are consistent with RT-PCR analysis. We tested for expected tissue-specific expression of 107 genes (a mixture of characterized and uncharacterized) in 18 selected tissues. In this analysis a single primer pair was tested for each gene. (It is possible that the predicted exon structures for many of the poorly characterized XM genes are incorrect: there was a clear correspondence between whether a product was obtained and whether there was an EST or cDNA in the public databases, which would indicate correct gene structures – see Materials and methods.) Among the 55 primer pairs that could result in amplification, 53 (96%) gave a correct-product size in the tissue(s) expected on the basis of our array data, and 47 (85%) produced amplification most strongly or exclusively in the expected tissue(s) (Figure 2b and data not shown). Although RT-PCR is semi-quantitative, there is an obvious correspondence between the left and right panels in Figure 2b, confirming that our microarray measurements are largely consistent with a more conventional expression analysis method.

Fourth, in the analyses detailed in the following sections, we show that the annotations of genes expressed preferentially in each tissue correspond in many cases to known physiological functions of the tissue, further confirming the accuracy of the dissections and the microarray measurements. Moreover, sets of functionally related genes were often observed to display uniform expression profiles, a result that is highly unlikely to occur by chance.

### Definition of 21,622 confidently detected transcripts

**Figure 3:**
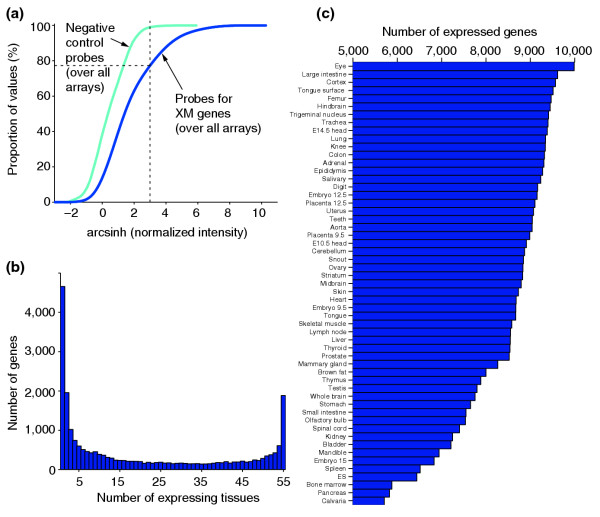
Defining whether a gene is expressed, and how many genes are detected as expressed per sample. **(a) **The curves show the cumulative distribution for negative-control probes (cyan line) and for probes on the array (blue line), over all arrays, to illustrate how genes were defined as expressed. The dotted black line indicates the 99^th^percentile for the negative control spots. **(b) **The number of genes expressed in any given number of tissues (between 1 tissue and 55 tissues; for example, there are 4,475 genes detected in only one sample, 171 genes expressed in exactly 27 samples, 1,790 genes detected in all 55 samples, and so on). The genes expressed in each of the 55 tissues were determined as in (a). **(c) **Number of genes defined as expressed in each of the 55 tissues, using criteria in (a).

**Figure 4:**
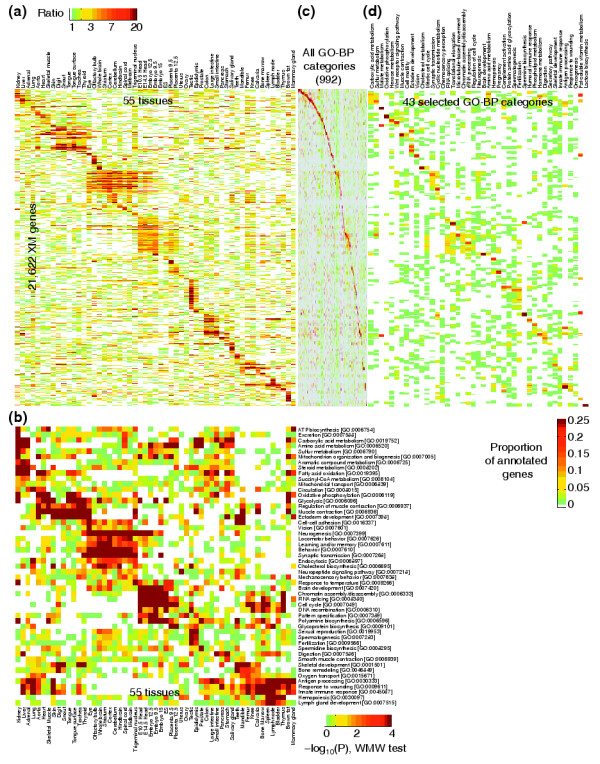
Correspondence between gene expression patterns and GO-BP annotations. **(a) **Ratios for the 21,622 expressed genes were grouped by two-dimensional hierarchical agglomerative clustering and diagonalization, using the Pearson correlation coefficient. **(b) **Negative logs of *P *values resulting from applying the Wilcoxon-Mann-Whitney (WMW) test to each of the GO-BP categories in each of the tissues are shown. The categories (vertical axis) were clustered and ordered as in (a). **(c, d) **'Density' of GO-BP annotations significantly enriched in specific points along the vertical axis at left (genes) are indicated; note that genes are in the same order in (a, b, c).

In order to establish rigorously which genes are expressed in each tissue sample, we used the 66 negative-control spots on our arrays (corresponding to 30 randomly generated sequences, 31 mouse intergenic or intronic regions, and five yeast genes). We considered the XM genes to be 'expressed' only if their intensity exceeded the 99th percentile (that is, all but 1%) of intensities from the negative controls (Figure 3a). 21,622 transcripts satisfied this criterion in at least one sample. There were 1,790 transcripts that were detected in every sample, and manual inspection verified that many of these have traditional 'housekeeping' functions (for example, ribosomal proteins, actin and tubulin). There were 4,475 transcripts detected in only one of the 55 samples (Figure 3b). Most of the 21,622 genes, however, were expressed in multiple tissues (Figure 3b). Each of the tissues expressed fewer than half of the 21,622 genes (Figure 3c). The number of genes detected in each sample was slightly lower than the conventional estimate of 10,000 genes expressed per cell (for example, we detected 6,094 different transcripts in embryonic stem (ES) cells, the only pure cell population examined, whereas a recent study using sequence tags indicated approximately 8,400 different transcripts in human ES cells [18]). This level of detection is not unexpected, for several reasons. First, tissues are mixtures of cell types, such that low-abundance, cell-type-specific transcripts may be diluted below the array detection limits of 1 in 1,000,000 [13]; second, the arrays did not include every single mouse gene; and third, our threshold for expression was conservative. The full 21,622 × 55 data matrix is found in the Additional data files. Figure 4a shows a clustering analysis of the 21,622 expressed genes in the 55 surveyed tissues, which illustrates that distinct tissues with related physiological roles also tend to have similar overall gene expression profiles. For example, all components of the nervous system featured higher expression of a common subset of transcripts, as did all components of the lower digestive tract.

### Correspondence between gene and tissue function

To examine the relationships among tissues and gene functions, we asked whether genes carrying specific Gene Ontology 'Biological Process' (GO-BP) categories, which reflect the physiological function of a gene, were preferentially expressed in each of the tissue samples, using a statistical test (Wilcoxon-Mann-Whitney; WMW). A selection of the WMW scores are shown in Figure 4b, and expression patterns of all genes in all GO-BP categories can be seen in the Additional data files and at the Toronto gene expressions website [19]. This analysis revealed that the preferentially expressed GO-BP categories typically reflected known functions of the tissue, sometimes with surprising resolution. For example, while the category 'synaptic transmission' scored highly in all neuronal tissues, 'learning and memory' was highest in cortex and striatum; 'locomotor behavior' was highest in cortex, midbrain, and spinal cord; 'response to temperature', in the trigeminal nucleus of the brainstem; and 'neurogenesis', in both adult central nervous system and embryonic heads (Figure 4d). While the WMW test may not have captured all of the categories relevant to each brain tissue, this finding does illustrate that our data contain differential expression of genes involved in distinct high-level neural functions. Further investigation of several tissue-associated GO-BP categories that were initially unanticipated revealed that they are easily rationalized; for instance, lung, bladder, skin, and intestines all express immune-related categories, presumably because they are exposed to the environment and infiltrated by immune cells (see for example, [20]).

### Correspondence between gene function and transcriptional co-expression

**Figure 5:**
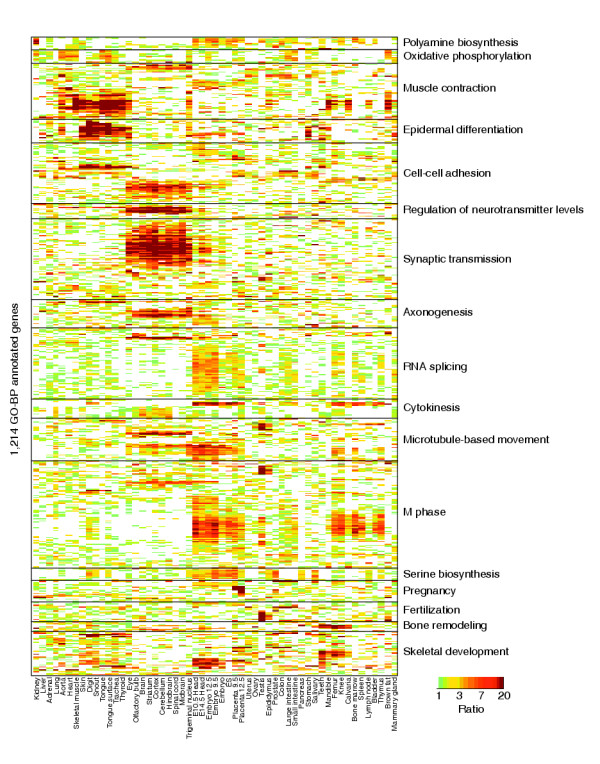
Expression of genes in 17 different functional categories. The categories were ordered manually. The genes within each category were clustered separately from those in other categories. The order of tissues is preserved from previous figures.

An alternative way to ask whether gene regulation corresponds to gene function is to examine the correlations among the transcript levels of genes, independent of the tissue-source information. An initial confirmation that patterns of transcript abundance correspond to gene functions comes from simply examining the behavior of all genes within distinct functional categories. For example, Figure 5 shows the expression of individual genes in 17 categories that exemplify ways in which gene expression relates to gene function (similar diagrams for all GO-BP categories can be seen in the Additional data files and at the Toronto gene expressions website [19]). There are prominent patterns that are distinctive of a subset of genes in each category. The fact that not all of the genes within each annotation category conformed to a single pattern could result from imperfections in the annotations or the measurements, or could be due to the correspondence between gene function and gene expression being less than absolute. While highly tissue-specific expression of genes in a category was observed in some cases (such as 'pregnancy' genes in placenta or 'fertilization' genes in testis), it was much more common that genes within a category were expressed across multiple functionally related tissues (for example, 'bone remodeling' in all bone tissues), consistent with the results shown in Figure 4b. In other instances, genes within a single annotation category were subdivided into multiple expression patterns: for example, 'cell-cell adhesion' contains three distinct groups of genes with elevated expression in skin-containing samples, neural tissues, and digestive tract, respectively. Consistent with a previous study [21], we observed coordinate regulation of genes within distinct biochemical pathways; Figure 5 includes the examples 'polyamine biosynthesis' and 'serine biosynthesis'. Moreover, a number of functional categories corresponding to basic cellular or biochemical functions which are traditionally thought of as 'housekeeping' (since they are required for cell viability) were in fact coordinately regulated across tissues: Figure 5 shows genes in the category 'RNA splicing', which are expressed most highly in neural and embryonic tissues, perhaps reflecting the higher levels of gene expression and alternative mRNA splicing known to occur in these tissues. Interestingly, subsets of genes in the categories 'cytokinesis', 'microtubule-based movement', 'oxidative phosphorylation', and 'M phase', all of which might be considered as central to cellular physiology, were also expressed in distinctive patterns among mouse tissues.

We also asked more generally whether groups of co-expressed transcripts were associated with specific GO-BP categories. Figure 4c shows that this is indeed the case: any given 'cluster' of genes with correlated expression levels is more likely than not to be associated with a local enrichment of one or a few annotation categories, and manual analysis suggests that tissue-specific expression often reflects the known physiological role(s) of the tissues in which the genes are expressed (examples are shown in Figure 4d). False-discovery rate analysis (see the Materials and methods section) confirmed that over 58% of the 21,622 genes were co-regulated with a set of genes significantly enriched for at least one GO-BP category. For the 7,387 GO-BP annotated genes, over 66% were co-expressed with a set of genes significantly enriched for at least one GO-BP category; in over 25% of these instances, the most significant category was one of its existing annotations. Random permutation analysis (that is, repeating the analysis with randomized gene identities) established a false discovery rate [22] of less than 1% for these analyses (see Materials and methods for details). Hence, quantitative co-expression of functionally related genes appears to be a general phenomenon in mammals.

### Using transcriptional co-expression to predict mouse gene functions

It stands to reason that a gene expressed in a specific tissue is likely to be functioning in that tissue. Therefore, we next asked how accurately mammalian gene functions can be predicted on the basis of gene expression profiles. There are many anecdotal examples in which the tissue-specific or cell-type-specific expression of a gene has been used to aid in discovering its function, and this approach has been advocated in previous analyses of mouse tissue expression data (see for example, [15]). Our data indicate that the expression of most mouse genes shows some degree of tissue restriction, but most of the genes are not expressed in a highly tissue-specific manner (Figure 3b). Furthermore, most tissues express genes from multiple functional categories (Figure 4b), and genes from many functional categories are expressed across many tissues (Figure 5), which could make it difficult to distinguish genes in these categories on the basis of expression in one or a few tissues. In addition, defining tissue specificity involves drawing thresholds to form lists, rather than using the quantitative expression information directly to draw functional inferences.

An alternative strategy is to generate functional predictions on the basis of transcriptional co-expression [23,24], which we show (above) often reflects gene function (Figure 5). This approach utilizes quantitative measurements and places no restriction on tissue-specificity, allowing all expressed genes to be treated equally in the analysis. Furthermore, the use of quantitative co-expression allows the application of sophisticated computational tools that have been optimized for the general problem of classification on the basis of features within a data matrix [25]. We examined the extent to which this approach is effective for our data, and we show (below) that it yields almost universally superior predictions of gene function in comparison to using information regarding simple tissue specificity or tissue restriction.

In this analysis, we used support vector machines (SVMs) [26]. An SVM is a machine-learning algorithm (a computer program) that has previously been shown to work well for the prediction of gene functions in yeast on the basis of microarray expression data [25] but which has not, to our knowledge, been used extensively to predict gene functions from mammalian expression-profiling data. The theory and implementation of SVMs have been described elsewhere in detail [25,26]. Briefly, an SVM outputs a 'discriminant value' for each gene in each category, and this value reflects relative confidence that the gene is in the category in question. The SVM considers each functional category separately, and the discriminant value is assigned on the basis of where the gene lies relative to other genes within the 'gene expression space' (for example, analysis of 55 samples results in 55 different coordinates). If the gene lies in a region where there is a high proportion of genes that are known to be in the category in question, this will lead to a high discriminant value. SVMs are conceptually related to clustering analysis in the sense that the discriminant values are derived from similarity among expression profiles. But in clustering analysis, genes are grouped solely on the basis of their expression levels; in contrast, SVMs use the known classifications (that is, knowledge regarding which genes are in the category and which are not) in order to map the initial gene expression space into a one-dimensional space (the discriminant values) in which the two classes are optimally distinguished.

Importantly, the discriminant values output by an SVM can be processed to obtain an estimate of the probability that the prediction for each gene in each category is correct (that is, an estimate of precision), on the basis of how well previously annotated genes in the given category can be distinguished from previously annotated genes that are not in the category. This is accomplished by a three-fold cross-validation strategy, in which the analysis is run three times, each time with a different one-third of the annotations masked so that the SVM algorithm does not know whether or not they are in the category when it is assigning discriminant values. Any given discriminant value is then converted to a precision value by simply asking what proportion of the masked genes with discriminant values above the given discriminant value really are in the category in question. The proportion of known genes in the category that are identified by the SVM as being in the category is also obtained at each discriminant value, and is referred to as recall. For all subsequent analyses we used precision and recall as our primary measures of success.

**Figure 6:**
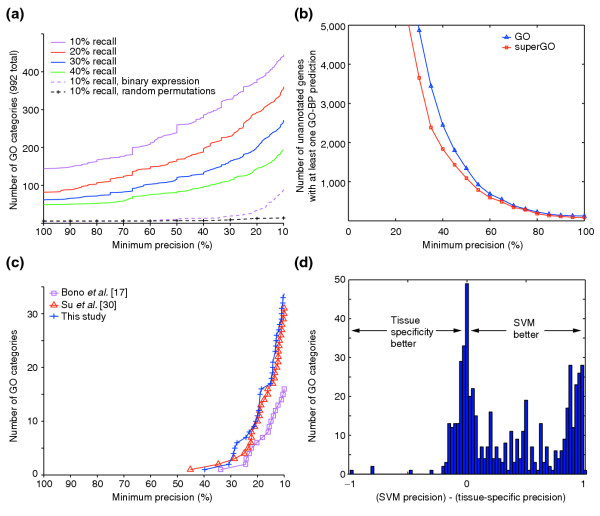
Predicting GO-BP categories of mouse genes using microarray data and SVMs. **(a) **The number of the 992 initial GO-BP categories exceeding the indicated precision value, with recall fixed for each line; for example at 40% recall (green line), around 100 categories achieve precision of 30%. To estimate the significance of the colored lines, we repeated their calculation after permuting the gene labels in the annotation database. The dotted gray line indicates the maximum number of GO categories that achieve the indicated precision, with recall of 10% or greater. The dotted magenta line indicates the result obtained using 'binary' expression data (expressed/not expressed) in each tissue. **(b) **The number of genes with predicted GO-BP categories (blue line) or superGO categories (red line) at varying precision values. The individual predictions are given in the Additional data files. **(c) **Comparison of the overall predictive capacity of three data sets, restricted to the 13 tissues and 1,800 genes shared by all three data sets. Each of the lines corresponds to the 30% recall line in (a). All of the lines are to the lower right of those in (a), since fewer genes and tissues were used. **(d) **A histogram comparing the precision of predictions derived from lists of tissue-specific genes with the precision of predictions from SVMs. For each category, the tissue-specific list yielding the highest precision value was identified, along with its associated recall value, and the SVM precision for the same category at the same recall value was identified. The difference between the two precision values is plotted for each category, such that instances where the SVM is superior are to the right of center.

We trained separate SVMs for each of the 992 GO-BP categories. This revealed that genes in hundreds of categories could be recognized with precision greater than 50% (Figure 6a). Typically, not all of the genes in a category could be recognized (the curves in Figure 6a correspond to recall of 10% through 40%); this is due to the fact that not all genes within any given category display the characteristic expression pattern (Figure 5). As a control, when the gene labels were randomized, only zero to fifteen categories (depending on the randomization run) achieved 10% precision and 10% recall simultaneously (black dotted line at the bottom of Figure 6a). Therefore, this analysis demonstrates that, in a blind test, the known genes in many functional categories can be distinguished on the basis of the expression profiles of other genes that are members of the same functional category. This implies that there are distinct regulatory mechanisms that control these pathways, and indicates that correlation-based methods can be used to predict the functions of uncharacterized genes in mammals.

### Predicted functions for unannotated genes are supported by sequence features

We next used these trained SVMs (Figure 6a) to predict functions for the 12,123 unannotated genes for which we detected expression in our data. The number of genes with at least one predicted function (that is, one GO-BP category) is shown in Figure 6b at varying precision thresholds (blue line). All of the predictions with precision above 15% are listed in the Additional data files. To make the outputs easier to peruse manually, we grouped 587 GO categories into 231 'superGO' categories, by combining categories that resulted in the same set of predicted genes and that were manually verified to be physiologically related. Figure 6b (red line) confirms that the number of unannotated genes that are predicted to have some function by an SVM with 'superGO' categories are similar to those with the original GO categories, although the number of categories has been compressed.

**Figure 7:**
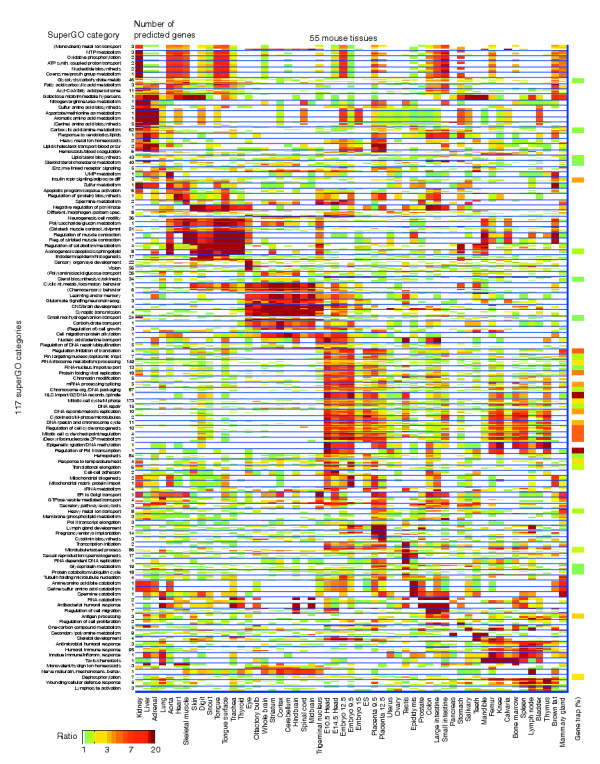
Expression patterns of 1,092 unannotated genes predicted to belong to any of 117 'superGO' categories with 50% confidence or higher. The vertical axis was clustered and diagonalized as in Figure 4. The height of each predicted category has been normalized to facilitate display; the number of genes predicted in each category is indicated at the left. The gene order (vertical axis) has been clustered within each category to illustrate that some categories are characterized by multiple patterns. The proportion (%) of predicted genes in each category that have gene-trap ES cell lines available are represented at right (color scale from 0 to 100%).

In order to provide a set of 'highest priority' predictions, we singled out those with the highest estimated precision. Among the unannotated genes (that is, those carrying no annotation in GO-BP), 1,092 (representing 117 superGO categories) were associated with precision values of 50% or greater; thus, on the basis of the analysis above, each of these genes is more than 50% likely to be involved in the given biological process. Figure 7 shows the original microarray data for these 1,092 genes, sorted by the predicted categories. Predictions were made for genes expressed in all of the tissues analyzed, and represent a wide spectrum of biological processes.

While some predictions correspond to expression in a single tissue (for example, the 56 genes predicted in 'vision' were predominantly expressed in the eye), such cases were unusual. Rather, most of the predictions were based on expression in multiple functionally related tissues (for example, the five genes predicted in 'regulation of cell migration' were characterized primarily by high expression in colon, large intestine, and small intestine) or more complex patterns (for example, genes predicted in 'CNS/brain development' were preferentially expressed in all adult neural tissues as well as in embryonic heads). Many predictions were found to be in categories related to the cell cycle and RNA processing. These genes tended to be expressed constitutively, but were most highly expressed in embryonic tissues, presumably because of rapid cell growth during development. However, many other predictions relate to neural functions, the immune response, muscle contraction, small-molecule metabolism, and other aspects of adult physiology. All of the individual predictions are provided in a table in the Additional data files, together with the expected precision and other information regarding the gene and the encoded protein, and these can be sorted by gene or by functional category.

Among the 1,092 unannotated genes, 488 (45%) have no overt sequence features suggesting physiological or biochemical function (that is, they have no similarity to previously characterized proteins or known functional domains; they are listed in Additional data files; and also see Materials and methods). Examination of the remaining 55% provided evidence that many of the predictions are likely to be correct. First, a handful of genes that were not annotated in our version of GO have in fact been characterized in the literature. For example, SVMs correctly predicted that phospholamban, the regulator of the Ca^2+^-ATPase in cardiac sarcoplasmic reticulum [27] is involved in 'muscle contraction or development'. Other genes are similar to characterized genes in other species: for example, the mouse homolog of the yeast 'Extra Spindle Poles' (*ESP1*) gene was predicted by SVM to function in 'mitotic cell cycle', 'cytokinesis', and 'microtubule based process', consistent with the function of its yeast counterpart [28].

A more comprehensive and objective analysis was enabled by the fact that, in an independent sequence-based analysis we conducted (see Materials and methods), known protein domain structures were encoded by 461 (42%) of these 1,092 unannotated genes (listed in the Additional data; see also the Materials and methods section) [29]. These provided further independent support for many of the predictions, since neither the primary sequences nor the domain features of the unannotated genes played a part in the predictions. In many cases, the domains also augment the predicted physiological function with a potential biochemical mechanism. For example, 3 of the 11 genes predicted in the category 'acyl-CoA/fatty acid/peroxisome' encode a short-chain dehydrogenase motif, suggesting that they are metabolic enzymes. Among the 86 unannotated genes predicted to function in 'microtubule-based process' are 4 with chromosome-segregation ATPase domains, one with an intermediate filament protein domain, one with a kinesin-motor domain, one with a myosin heavy-chain domain, and one with a tropomodulin domain, all of which are consistent with microtubule- and/or cytoskeleton-related functions. Of the four proteins predicted in 'skeletal development', one encodes a fibrillar collagen carboxy-terminal domain, and one encodes a collagen triple-helix repeat. Some of the relationships between predictions and domains are striking on the simple basis of their numbers: 7 of the 95 genes predicted in 'humoral immune response' encode an immunoglobulin domain; 13 of the 87 genes predicted in 'chromosome organization/DNA packaging' have high mobility group (HMG) domains, and 23 of the 149 genes predicted in 'RNA processing/ribosome biogenesis' encode helicase domains, RNA-binding domains, or RNA-modifying motifs. Table 1 lists a selection of statistically significant associations between the different prediction classes shown in Figure 7 and protein domains.

### Comparisons among data sets for predicting gene functions

Although there was a significant correlation among the three different mouse tissue-specific data sets compared in Figure 2a, there were also many cases in which the three data sets disagreed in their assessment of relative abundance of individual genes in different tissues (Figure 2a and data not shown). We reasoned that the SVM cross-validation analysis could provide an objective measure of the quality of the different data sets: since random measurements lead to very poor predictions (Figure 6a), any errors in the data would tend to degrade the precision and recall values. While our manuscript was in preparation, an additional data set was released by Su *et al.*, the authors of reference [15]. Their newer data [30] include measurements of 36,182 known and predicted genes over 61 tissues, measured in duplicate using custom-built Affymetrix arrays, and are thus similar in scope to our data set. Figure 6c shows a comparison between cross-validation results from running SVMs on the three data sets: ours, that of Su *et al. *[30], and that of Bono *et al. *[17], with each restricted to the 13 tissues and 1,800 genes common to all three, and the same GO-BP annotations used for all three data sets. Figure 6c shows that, although our data fare slightly better, the power of our data set and that of Su *et al. *[30] for predicting GO-BP categories are comparable. This confirms that distinct and coordinate regulation of many mammalian functional pathways is authentic because it is observed in two independent data sets.

### Comparison of tissue-specificity with co-expression for predicting gene functions

We used two different approaches to ask how well tissue specificity can predict the functional classes of genes, in comparison to co-expression. First, from our data we compiled three sets of lists: genes that are expressed in each of the 55 individual samples; genes that are expressed highest in each of the individual 55 samples and in groups of functionally related samples (for example, treating all neural tissues as a single group); and also genes that are expressed uniquely in individual samples. All of these lists (175 in total) are compiled in the Additional data files. For each of the 992 GO-BP categories, we assessed the precision and recall for each of these lists (that is, whether these lists can distinguish genes in the category from those not in the category), and then identified the best precision value and its associated recall value for that category. Figure 6d shows a histogram of the difference between SVM precision and tissue-specificity precision, at the same recall value, for each GO-BP category. The vast majority of data points are greater than zero (*P *< 10^-76^; two-sided pairwise *t *test), indicating that co-expression patterns can be used (by SVMs) to predict gene functions significantly better than tissue-specificity alone.

It is possible that improved results might be obtained by other *ad hoc *procedures for sorting the genes in different ways, or by more automated procedures for generating large numbers of lists. However, an alternative analysis suggests that this is unlikely: when we re-ran SVMs with the matrix of 1s and 0s indicating which gene is expressed (or not) in each tissue, rather than the matrix of quantitative expression values, the resulting predictions were inferior (dotted magenta line in Figure 6a). In theory, if any combination of on/off information about gene expression in different tissues was informative for identifying genes in any category, it would have been identified by the SVMs. The result we obtained indicates that the quantitative measurements contain critical information reflecting functions of genes that is not, for the most part, contained in the binary (expressed/not expressed) information.

### Validation of predictions by *de novo *functional analysis

**Figure 8:**
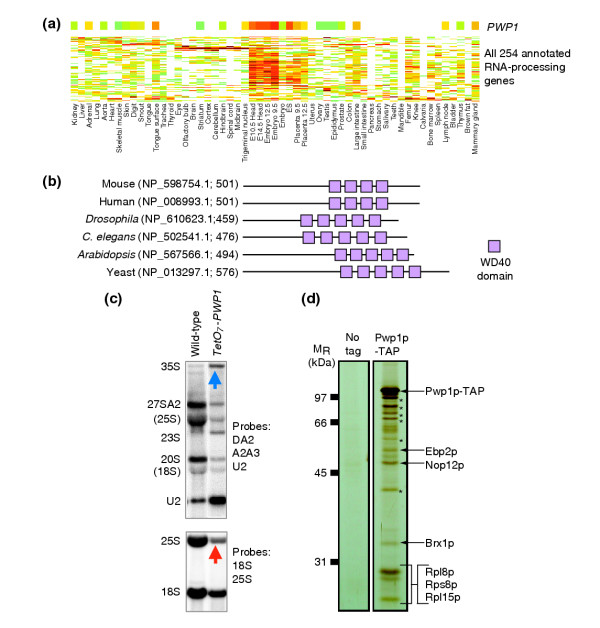
*PWP1 *functions in ribosomal large-subunit biogenesis. **(a) **The expression pattern of mouse Pwp1 is similar to that of most known RNA-processing proteins. **(b) **The domain structures of Pwp1 homologs identified by BLASTP searches. Accession number and amino-acid length is given. We identified a single strong match in each of the species shown. Domains were identified by CDD search [29]. **(c) **A northern blot showing the accumulation of 35S rRNA precursor (blue arrow), reduction in other rRNA precursors (top panel), and reduction in 25S rRNA (red arrow) in the yeast *TetO*_7_-*PWP1 *mutant (strain TH_2220) in comparison to the parental wild-type strain (R1158) [9]. The U2 spliceosomal RNA is shown for comparison; its apparent abundance is increased because 5 μg RNA was loaded per lane, and the relative proportion of rRNA to snRNA is decreased in the mutant. Blotting procedures and probes were as previously described [9]. **(d) **Affinity-purification of yeast Pwp1p-TAP reveals association with proteins known to function in ribosomal large-subunit biogenesis (Ebp2p, Nop12p, Brx1p) as well as a subset of ribosomal proteins. The asterisks mark degradation products of Pwp1p-TAP.

Finally, we asked whether new functional predictions could be confirmed by directed experimentation. Among the genes we predicted to function in RNA processing and ribosome biogenesis was *PWP1*, which encodes a protein that includes WD40 repeats and which has previously been found to be up-regulated in pancreatic cancer tissue [31]. In our data, *PWP1 *was most highly expressed in embryonic tissues, as is characteristic of most genes annotated as 'RNA processing' by GO-BP (Figure 8a). The encoded protein Pwp1p is highly conserved across eukarya (Figure 8b) but to our knowledge it has not been functionally characterized in any species, although it has been found in the human nucleolus [32], and in yeast it is essential for cell growth [33]. We created a titratable-promoter allele of yeast *PWP1*, and found that cells depleted for Pwp1p displayed a striking reduction in 25S rRNA (Figure 8c), confirming the involvement of this gene in RNA processing and ribosome biogenesis. Given that WD40 repeats are thought to be protein interaction domains, we also asked whether Pwp1p physically associates with other proteins. We found that epitope-tagged yeast Pwp1 protein co-purified with known *trans*-acting ribosome biogenesis factors, as well as with several ribosomal protein subunits (Figure 8d), consistent with a direct role in ribosome biosynthesis.

## Discussion

### Simultaneous gene discovery, network mapping, and functional inference

The data presented here and the resulting inferences for the physiological roles of mammalian genes significantly extend previous microarray-based analyses of mammalian gene expression [7,15,17,21,23,24,30]. First, the data support the notion that there are thousands of mouse genes that are not represented in current cDNA databases [12,34-36]. Amongst all 21,622 confidently detected transcripts (Figure 4), 5,600 were not associated with a cDNA; 3,551 of these had EST but not cDNA support, indicating that many of them correspond to *bona fide *genes (Figure 2b). Moreover, inferences for the physiological roles of these transcripts can be obtained by analysis of quantitative expression levels across tissues; the 1,092 unannotated genes for which we made high-confidence predictions (Figure 7) contain 54 with no EST or cDNA support, and an additional 114 that have only EST support.

Second, our estimate that more than 58% of all transcripts are regulated together with genes in specific functional categories is much higher than previous estimates. One analysis, based on cursory analysis of early yeast and *Xenopus *expression data, suggested that only 5–10% of all genes fall within 'synexpression' groups [2]. Our results represent a minimum estimate of the correspondence between gene function and gene expression, because shortcomings in either the annotations or the data would tend to reduce these figures. Our results indicate that there are regulatory pathways that control many distinct biological processes in mammals, and that it is already possible to interpret the expression patterns of the majority of mammalian genes in a functionally meaningful way by comparing them to the patterns of the subset of genes that are already annotated. Moreover, it may be more straightforward than was previously anticipated to apply the same computational techniques to mammalian microarray data that are now being applied to identify 'network modules' and regulatory mechanisms in far simpler organisms, such as yeast (see for example, [37]). These potential applications represent an obvious future extension of the work presented here.

Third, while previous analyses using microarray expression data to predict gene functions [1-10] have focused on the fact that genes in large or general categories can be recognized (for example, ribosome biogenesis, translation, or proteolysis), we show quantitatively that this methodology is applicable to a much wider variety of functional categories, many of which are specific to higher organisms (for example, the category 'Pregnancy/embryo implantation' is specific to mammals; Figures 5,7).

Fourth, our analysis shows that the use of quantitative gene expression measurements to infer mammalian gene functions is more powerful than the traditional approach of using information on simple tissue specificity. Genes in many GO-BP categories were precisely identified by SVM using quantitative co-expression, but not on the basis of tissue specificity or tissue restriction (Figure 6a,6d). It appears from our results that genes in functional categories with more widespread expression (such as 'epidermal differentiation', 'regulation of cell migration', and 'apoptotic program'), categories corresponding to basic cellular functions (such as 'oxidative phosphorylation', 'RNA processing', and 'DNA replication'), and even categories that describe interactions among different cell types ('taxis', 'glycoprotein metabolism', 'cell-cell adhesion') can all be recognized and distinguished from genes in other categories on the sole basis of their coordinate expression across many tissues (Figures 5,7), even though they are expressed in many tissues (and in some cases all tissues, as in the case of mRNA splicing and other 'housekeeping' functions). *PWP1 *is an example of a gene that is widely expressed but has a pattern of expression that was predictive of its function (Figure 8).

### A strategy and resource for mouse functional genomics

Analysis of mutant phenotypes is one of the most powerful and definitive ways to study gene functions. Many of the predicted gene functions (see Figure 7 and the Additional data files) in turn predict specific mutant phenotypes; for example, mutation of genes predicted to function in 'vision' would be expected to display defects in sight or eye morphology, while mutation of those predicted to function in 'RNA processing/ribosome biogenesis' might be lethal embryonically, but with alterations in RNA profiles or ribosome content. We have already initiated efforts to validate predicted gene functions in animals: of the XM genes on our array, 2,917 are already represented in collections of publicly available gene-trap ES cell lines [38] (indicated on the right in Figure 7). It will become increasingly straightforward to test these predictions as RNA interference methods are refined and the collection of mapped mouse mutants expands [38-40]. All of our predictions are listed in the Additional data files and will provide guidance for future efforts in mammalian functional genomics and/or support for other functional studies.

In contrast to other available mouse tissue-specific data sets (such as those described in [30]), all of our data, as well as the SVM predictions, can be downloaded anonymously without restriction from the Additional data files or from our website [16] and can be freely copied, modified, and propagated. The oligonucleotide sequences are provided, so that copies of our array design can be obtained and modified by other labs, and our expression data can be mapped to any clone collection or updated sequence annotation by batch BLAST. Many other supporting files are provided, including GO annotations, maps to gene-trap collections, and genomic locations of the probes. To facilitate perusal of the data, we have also created a web tool [19] that displays subsets of the gene expression data together with functional information and SVM predictions. This tool currently supports queries originating with a gene of interest, a functional category of interest, or a region of the chromosome of interest, which may facilitate the use of gene expression patterns and predicted gene functions in identifying genes that confer mapped traits [41]. We anticipate expanding and refining this resource to mirror both additional data and updated annotations.

## Conclusion

We have created an extensive mouse expression data set and asked whether quantitative gene expression patterns correspond to functional gene categories. Our major finding is that most tissues express many functional categories, consistent with the fact that they contain many different cell types performing many different functions, but that many different functional pathways are coordinately expressed in a quantitative manner across tissues such that many categories display one or more distinctive patterns. For example, embryonic heads contain many cell types, and consequently express genes in a variety of categories including 'CNS/brain development', 'M phase', 'skeletal development', and 'microtubule-based process', yet an SVM can distinguish genes in these categories because they are differentially regulated across all 55 tissues in a way that is characteristic for each functional category (Figures 5,7). The simplest explanation for this observation is that there are discrete factors or sets of factors that control each coordinately regulated pathway. We conclude that functional genomics strategies that rely on quantitative transcriptional co-expression will be as fruitful in mammals as they have been in simpler organisms, and that transcriptional control of mammalian physiology may be more modular than is generally appreciated.

## Materials and methods

### Mouse mRNA isolation

Mouse tissues were isolated from the following strains: ICR (whole brain, testis, skeletal muscle, heart, lung, liver, embryo at 15 days, embryo at 12.5 days, embryo at 9.5 days, mammary gland, placenta at 9.5 days and placenta at 12.5 days); CD1 (Charles River Laboratory, Wilmington, USA; cortex, cerebellum, striatum, hindbrain, midbrain, bone marrow, knee, teeth, mandible, calvaria, femur (bone marrow flushed diaphyses), tongue surface, snout, large intestine, thyroid, aorta, brown fat, lymph node, olfactory bulb, adrenal gland, prostate, digits, trachea, trigeminal nucleus); C3H (The Jackson Laboratory, Bar Harbor, USA; salivary gland, thymus, ovary, uterus, tongue, stomach, small intestine, spleen, colon, uterus, pancreas, epididymis, eye, bladder, skin); C57BL/6 (The Jackson Laboratory, Bar Harbor, USA; spinal cord); Black Swiss (NTac:NIHBS; Embryonic heads); and R1 (ES cells). With the exception of embryonic tissues and ES cells, tissues were harvested from 3–6 month-old mice. Following recommended University of Toronto protocols, mice were euthanized by barbiturate injection and tissues were dissected as quickly as possible (within 10 minutes), snap-frozen in liquid nitrogen, and preserved at -80°C until use. RNA was extracted using homogenization and Trizol reagent (Invitrogen, Carlsbad, USA) following the instructions from the manufacturer, and mRNA was purified as described previously [3].

### Microarray probe design

A FASTA file of 42,192 known and predicted mRNAs (XM sequences) was obtained from Deanna Church at NCBI on July 9, 2002 and is posted as Additional data file 1. Interspersed repeats and low complexity DNA sequences were masked with Repeat-Masker [42]. The 500 nucleotides from the 3' end of each mRNA were extracted and 10 non-overlapping T_m_-balanced probes were generated using PrimerX [43] with default settings. The most unique among the 10 was identified on the basis of having the highest ΔG difference between the first (identical) and second most significant BLAST hits among the 42,192 initial XM mRNAs. Then, 41,699 probe sequences (those for which probes could be designed using this procedure) were submitted for oligonucleotide microarray production (Agilent Technologies, Palo Alto, USA). These arrays are manufactured using an ink-jet process, in which oligonucleotides are synthesized on the array by direct deposition of phosphoramidites [13]. The specificity, sensitivity, and reproducibility of these 60-mer arrays has been described elsewhere in detail [13].

Among the probes on the array, 40,822 were unique; those that were not unique can be attributed primarily to gene duplications, predominantly pseudogenes of GAPDH, ribosomal proteins, and retrovirus-like sequences. To minimize the impact of redundancy on statistical analyses, we collapsed the data from 1,928 duplicated probes and XM sequences that were in these sequence families (including 100 probes duplicated between the two array designs) into 525 groups that shared identical probe sequence and/or were both annotated and regulated in the same way. We also mapped all of the XM sequences to the current version of the mouse genome (Build 32) and to three cDNA databases (UniGene, RefSeq, and Fantom II; see below) and identified 1,991 XM sequences in which XM sequences adjacent on the chromosome also mapped to the same cDNA; these were collapsed into 904 groups. The Additional data files include a table mapping the 41,699 probes against the 39,309 presumed distinct transcripts.

### Labeling and hybridization

The mRNA (1–2 μg) was reverse-transcribed with random nonamer primers (1 μg per reaction) and T_18_VN (0.25 μg per reaction) to synthesize cDNA. The reaction contained a 1:1 mixture of 5-(3-aminoallyl) thymidine 5'-triphosphate (Sigma, St. Louis, USA) and thymidine triphosphate (TTP) in place of TTP alone. The cDNA products were bound to QIAquick PCR Purification columns (Qiagen, Hilden, Germany) following the manufacturer's instructions, washed three times with 80% ethanol, and eluted with water. Purified cDNA was reacted with N-hydroxy succinimide esters of Cy3 or Cy5 (Amersham Pharmacia Biotech, Piscataway, USA) following the manufacturer's instructions. Hydroxylamine-quenched Cy-labeled cDNAs were separated from free dye molecules using QIAquick columns. Mixed labeled cDNAs were added to hybridization buffer containing 1 M NaCl, 0.5% sodium sarcosine, 50 mM methyl ethane sulfonate (MES), pH 6.5, 33% formamide and 40 μg herring sperm DNA (Invitrogen, Carlsbad, USA). Hybridizations were carried out in a final volume of 0.5 ml injecting into an Agilent hybridization chamber at 42°C on a rotating platform in a hybridization oven (Robbins Scientific Corporation, Sunnyvale, USA) for 16–24 h. Slides were then washed (rocking for approximately 30 seconds in 6 × SSPE, 0.005% sarcosine, then rocking for approximately 30 seconds in 0.06 × SSPE) and scanned with a 4000A microarray scanner (Axon Instruments, Union City, USA). Hybridizations were performed in duplicate with fluor reversal: that is, each mRNA sample was examined in duplicate, once in the Cy3 channel and once in the Cy5 channel, on separate arrays. Each array was hybridized with two samples simultaneously, each from an individual tissue. Essentially identical results were obtained from single-channel data from the same mRNA sample analyzed on different arrays, which were distinct from individual channels on the same arrays analyzed with a different mRNA. The organization of the hybridizations, and the data for individual channels, are given in the Additional data files.

### Image processing and normalization

TIFF images were quantitated with GenePix (Axon Instruments). Individual channels were spatially detrended (that is, overall correlations between spot intensity and position on the slide were removed) by high-pass filtering (see [44]) using 10% outliers. We applied variance stabilizing normalization (VSN) [45] using 25% of the genes to normalize all single channels to each other. We manually identified and removed measurements that were inconsistent between dye-swaps, by either removing data from residual artifacts apparent on microarray images or removing the higher of the two disparate intensity measurements (in order to minimize false-positive detections). Measurements were transformed to arcsinh values (which are similar to natural log values, but are defined for negative numbers which emerge from the VSN) and for each measurement the median across all arrays was subtracted to obtain relative expression ratios for each gene in each tissue compared to all tissues. Remaining inconsistencies between dye-swaps were addressed by removing the higher of any two measurements that differed by more than two arcsinh units (in order to further minimize false-positive detections). The dye-swap arcsinh values were then averaged between replicates and among multiple probes detecting the same sequence. Clustering and manual analysis indicated that ratios below zero were generally not biologically meaningful (and probably stem largely from measurement error among low-intensity spots); hence ratios below zero were set to zero for all analyses using median-subtracted arcsinh values (Figures 1, 2, 4, 5, 6, 7 and SVM analyses). Missing values (fewer than 0.01% of all data points) were set to zero. Median-subtracted arcsinh values correspond approximately to the following ratios (arcsinh = linear): 0 = 1/1; 1 = 2.7/1; 2 = 7.5/1; 3 = 20/1, 4 = 55/1; 5 = 155/1, 6 = 405/1.

### Annotations

Mouse GO-BP annotations were downloaded from the Gene Ontology website [46] and the European Bioinformatics Institute (EBI) [47] and both were mapped to XM gene sequences by sequence identity to the annotated source sequences. The full annotation database is on our website [16]. Fewer than 0.01% of these annotations were derived from gene expression (IEP code); we confirmed that removal of these genes had no appreciable impact on statistical analysis or the SVM analysis, and hence the use of these annotations to analyze gene expression is not circular. The Mouse Genome Informatics (MGI) annotations are reported to be manually compiled, whereas the EBI annotations include automated sequence-based annotations (for example, potassium channels are annotated as being in 'ion transport' and the mouse homolog of the yeast Tim8 protein, which is a translocase of the inner mitochondrial membrane, is annotated as being in 'mitochondrial translocation'). All GO-BP annotations were propagated up all possible edges of the GO graph. Redundant GO-BP categories were excluded. Categories with fewer than three genes among the 21,622 expressed genes were excluded from our analysis since they are not appropriate for the statistical tests we used, and those with more than 500 genes were excluded because they are not specific to distinct physiological processes.

### False-discovery rate analysis

Each gene was associated with a co-regulated group consisting of the 50 annotated genes with the highest Pearson correlation coefficient relative to it. Annotation enrichment of this group in each GO-BP category was scored using the hypergeometric *P *value [48]. The minimum value of this score across all GO-BP categories was used as the measure for significant enrichment in any GO-BP category. *P *values were assigned to these measures using a permutation scheme on the gene labels. The statistical significance of the *P *values was evaluated using the Benjamini-Hochberg (BH) linear step-up procedure [22] to ensure a false discovery rate (FDR) of less than 1%. For annotated genes, a second measure was computed: the minimum among its annotated categories of the hypergeometric *P *values of its co-regulated group. A gene-specific permutation scheme associated *P *values with these scores and the FDR was also controlled at 1%.

### Cluster diagonalization

Starting with an initial hierarchical clustering (agglomerative, average linkage, based on Pearson correlation coefficient), rows were divided into groups by removing a small number of links at the highest levels of the tree and grouping together all rows contained within the same disconnected subtree. Each row group was then associated with the column that contained the maximum expression value averaged over all the profiles in the group. The row groups were then sorted in increasing order of their associated column numbers.

### Support vector machines

We used the SVM software package Gist [49] version 2.0.8 in Linux with parameter settings '-radial -zeromeanrow -diagfactor 0.5'. Precision was established by three-fold cross validation.

### Identification of corresponding clones in cDNA and EST databases

We identified the closest corresponding mouse mRNAs in FANTOM II [50] (60,770 sequences); RefSeq [51] (16,601 sequences); UniGene [52] (87,495 sequences); and Ensembl [53] (32,911 sequences) using BLASTN with a threshold of E-60. We identified corresponding mouse mRNAs in dbEST [54] (3,939,961 sequences) using BLASTN with a threshold of E-20.

### Identification of genes common to other microarray data and Spearman rank correlations

For Figure 2a, mRNA sequences were downloaded from [55] (for Su *et al. *data [15]) and [56] (for Bono *et al. *data [17]). The Su *et al. *[15] gene expression data were downloaded from [57] (9,977 sequences represented on the array) and the Bono *et al. *[17] data, from [58] (54,005 sequences represented on the array). The selected 41,699 NCBI mRNAs were used in a BLAST search against these two mRNA databases; a BLAST comparison between the two databases was also performed to retain only genes for which the closest sequence to each XM gene is also the closest sequence between the two other databases. All BLAST searches were performed with threshold E-60, and the best hit was selected for the multiple blast results. The 1,109 genes that have common hits in all the BLAST results and with gene expression data available were selected for the gene expression analysis. The 1,109 genes from all three datasets were normalized to make them comparable. To facilitate comparison, in the Bono *et al. *[17] dataset, each gene was median-centered in each tissue by subtracting its median expression value across all 13 common tissues. The Su *et al. *[15] data were arcsinh-transformed before median-centering. The data from the study described here that was used in the comparison was not zeroed, as it was in other analyses, and was median-centered using the median calculated only on the 13 common tissues, rather than all 55. The Spearman rank correlation coefficients of each pair of tissues among all three studies were transformed to Z-scores by multiplication by sqrt(1108) and then converted to *P *values using the cumulative probability density of a standard normal distribution.

For Figure 6c, an alternative mapping strategy was employed: our probe sequences, the Bono *et al. *[17] clone sequences, and the Su *et al. *[30] probe sequences were associated with 30,832 MGI sequences by mapping directly to corresponding MGI/GenBank sequences; 1,800 genes were identified in which a reciprocal best match between the probe sequences and the MGI sequence was identified in all three studies.

### RT-PCR

Primer pairs were designed to have a matching T_m _(59°C) and sequences are listed in the Additional data files. RT-PCR assays were performed using the OneStep RT-PCR Kit (Qiagen). Reactions were performed in 25 μl volumes containing 0.5 ng polyA^+ ^mRNA, 7.5 units porcine RNAguard (Amersham) and 300 pM each of the forward and reverse primers. After 30 rounds of amplification, the reaction products were separated on 2% agarose gels stained with ethidium bromide. Inverted black-and-white images of the gels were recorded using a Syngene gel documentation system and GeneSnap software (Synopics, Frederick, USA). In total, 107 primer pairs were tested. Of the 57 XM genes tested that corresponded to a known cDNA, 42 were among those that were amplified (74%). Of the 25 tested that corresponded to an EST but not to a known cDNA, 12 were amplified (48%). However, of the 25 tested that did not correspond to a cDNA or EST, only one was amplified (4%).

### Identification of genes associated with gene traps

Six different gene-trap resources were searched to identify genes associated with gene trap ES cell lines. For BayGenomics [59], Centre for Modeling Human Disease (CMHD) [60], University of California Resource of Gene Trap Insertions [61], and Fred Hutchinson Cancer Research Center (FHCRC) [62], the gene-trap sequence tags were downloaded from the website and searched against the selected 41,699 mRNA sequences using BLASTN. For the German Genetrap Consortium (GGTC) [63] and Mammalian Functional Genomics Centre (MFGC) [64], the web-based BLAST servers were used to search the 41,699 mRNA sequences against their gene-trap sequence databases. The hits with lengths equal to or larger than 50 nucleotides, and identity equal to or larger than 98%, were considered to be associated with the gene-trap ES-cell lines.

### RNA extraction, northern blotting, affinity purification, and mass spectrometry

The *TetO*_7_-*PWP1 *and isogenic wild-type control strains were created and analyzed as previously described for other essential yeast genes [9]. Briefly, strains were exposed to 10 μg/ml doxycycline (Sigma) for a total of 24 h before harvesting for RNA extraction. RNA extraction and northern blotting were performed using standard protocols and oligonucleotide probes as described previously [9]. TAP purification of Pwp1p was performed as previously described [9] using 1.3l culture volumes; gel-purified proteins were identified by MALDI-TOF mass spectrometry.

## Additional data files

There are 40 Additional data files comprising all the raw data; they are also available on our website [16]. A web tool for querying and browsing the data online is also available [19].

## Supplementary Material

Additional data file 1 XM (predicted mRNA sequences) from NCBI Click here for additional data file

Additional data file 2 XP (encoded protein sequences) from NCBI Click here for additional data file

Additional data file 3 Array Design 1 (spot map) Click here for additional data file

Additional data file 4 Array Design 2 (spot map) Click here for additional data file

Additional data file 5 Array Master file - 41,699 probes; the Master file contains probe sequences, columns listing the closest sequences in RIKEN, ENSEMBL, Refseq, and Unigene; GenBank description, EST overlap, GO-BP annotations, Domain (most significant) Click here for additional data file

Additional data file 6 Array information after removal of redundant probes: probe combinations Click here for additional data file

Additional data file 7 Array information after removal of redundant probes: master file - 39,309 presumed distinct transcripts Click here for additional data file

Additional data file 8 Hybridization records Click here for additional data file

Additional data file 9 Figure 1 data Click here for additional data file

Additional data file 10 Figure 2b data Click here for additional data file

Additional data file 11 Figure 4a data Click here for additional data file

Additional data file 12 Figure 4b data Click here for additional data file

Additional data file 13 Figure 4c data Click here for additional data file

Additional data file 14 Figure 4d data Click here for additional data file

Additional data file 15 Figure 5 data Click here for additional data file

Additional data file 16 Figure 7 data Click here for additional data file

Additional data file 17 Supplementary figures S1-3 Click here for additional data file

Additional data file 18 Data: 41,699 probes - single channel, arcsinh intensities Click here for additional data file

Additional data file 19 Data: 41,699 probes - replicates combined, arcsinh intensities Click here for additional data file

Additional data file 20 Data: 41,699 probes - median-subtracted, zeroed Click here for additional data file

Additional data file 21 Data: 39,309 presumed distinct transcripts - median-subtracted, zeroed Click here for additional data file

Additional data file 22 Data: 21,622 presumed distinct transcripts - median-subtracted and zeroed, expressed above 99% of negative-control spots Click here for additional data file

Additional data file 23 Data: binary matrix of expression above 99% of negative-control spots Click here for additional data file

Additional data file 24 Annotations: GO annotations among 39,309 presumed distinct transcripts (12,543 annotated genes, 47,900 annotations) Click here for additional data file

Additional data file 25 Annotations: GO annotations among 21,622 presumed distinct transcripts (9,499 annotated genes, 37,876 annotations) Click here for additional data file

Additional data file 26 Annotations: superGO annotations Click here for additional data file

Additional data file 27 Annotations: map between GO and superGO annotations Click here for additional data file

Additional data file 28 SVM Predictions: GO-BP SVM Predictions, 15% precision Click here for additional data file

Additional data file 29 SVM Predictions: GO-BP SVM Predictions, 50% precision Click here for additional data file

Additional data file 30 SVM Predictions: superGO SVM Predictions, 15% precision Click here for additional data file

Additional data file 31 SVM Predictions: superGO SVM Predictions, 50% precision Click here for additional data file

Additional data file 32 RT-PCR primer sequences Click here for additional data file

Additional data file 33 XM genes with gene trap lines Click here for additional data file

Additional data file 34 XM motifs Click here for additional data file

Additional data file 35 779 known and putative DNA-binding transcription factors among XM genes Click here for additional data file

Additional data file 36 Table of accession numbers of genes common to Zhang, Su, and Bono data Click here for additional data file

Additional data file 37 Tech report on spatial detrending Click here for additional data file

Additional data file 38 Map locations of XM genes (BLASTed against Build 32) - retained only if top hit of both XM sequence and array probe overlap (30,387 presumed distinct transcripts) Click here for additional data file

Additional data file 39 SVM functional predictions for 7,147 unannotated mapped transcripts (may be useful for positional cloning), also see [19] Click here for additional data file

Additional data file 40 175 lists of genes that are expressed in individual tissues, highest in individual tissues, or specific to individual tissues Click here for additional data file

## Figures and Tables

**Table 1:** Domains associated with genes predicted to function in specific biological processes

Predicted function	Enriched domain	Description of domain	Proportion of genes with this domain	-log_10 _significance (*P*)
Chromosome organization or DNA packaging	HMG	HMG (high mobility group) box	13/87	10.5
Pregnancy/embryo implantation	Hormone_1	Somatotropin hormone family	3/14	7.3
Acyl-CoA/fatty acid/peroxisome	FabG	Short-chain alcohol dehydrogenase	3/11	6.8
RNA/ribosome metabolism/processing	RRM	RNA recognition motif	10/149	6.6
Carboxylic acid/amine metabolism	ECH	Enoyl-CoA hydratase/isomerase family	3/62	6.2
Humoral immune response	Sp100	The function of this domain is unknown	2/95	6.1
Vision	Uteroglobin	The function of this domain is unknown	3/56	5.9
RNA-nucleus import/export	COG5136	U1 snRNP-specific protein C	2/13	5.7
Microtubule-based process	Smc	Chromosome-segregation ATPases	4/86	5.2

*P *values were calculated using the hypergeometric *P *value [48], which compares against expectation from random draws among the 15,443 XM genes with encoded domains. Domain names and descriptions are from the NCBI 'COG' database [65].
