# Supplementary material for: The functional landscape of mouse gene expression
Source: J Biol. 2004 Dec 6;3(5):21. doi: 10.1186/jbiol16 (PMC549719; doi:10.1186/jbiol16)
Supplement: Additional data file 17 — Supplementary figures S1-3 [file jbiol16-s17.pdf]

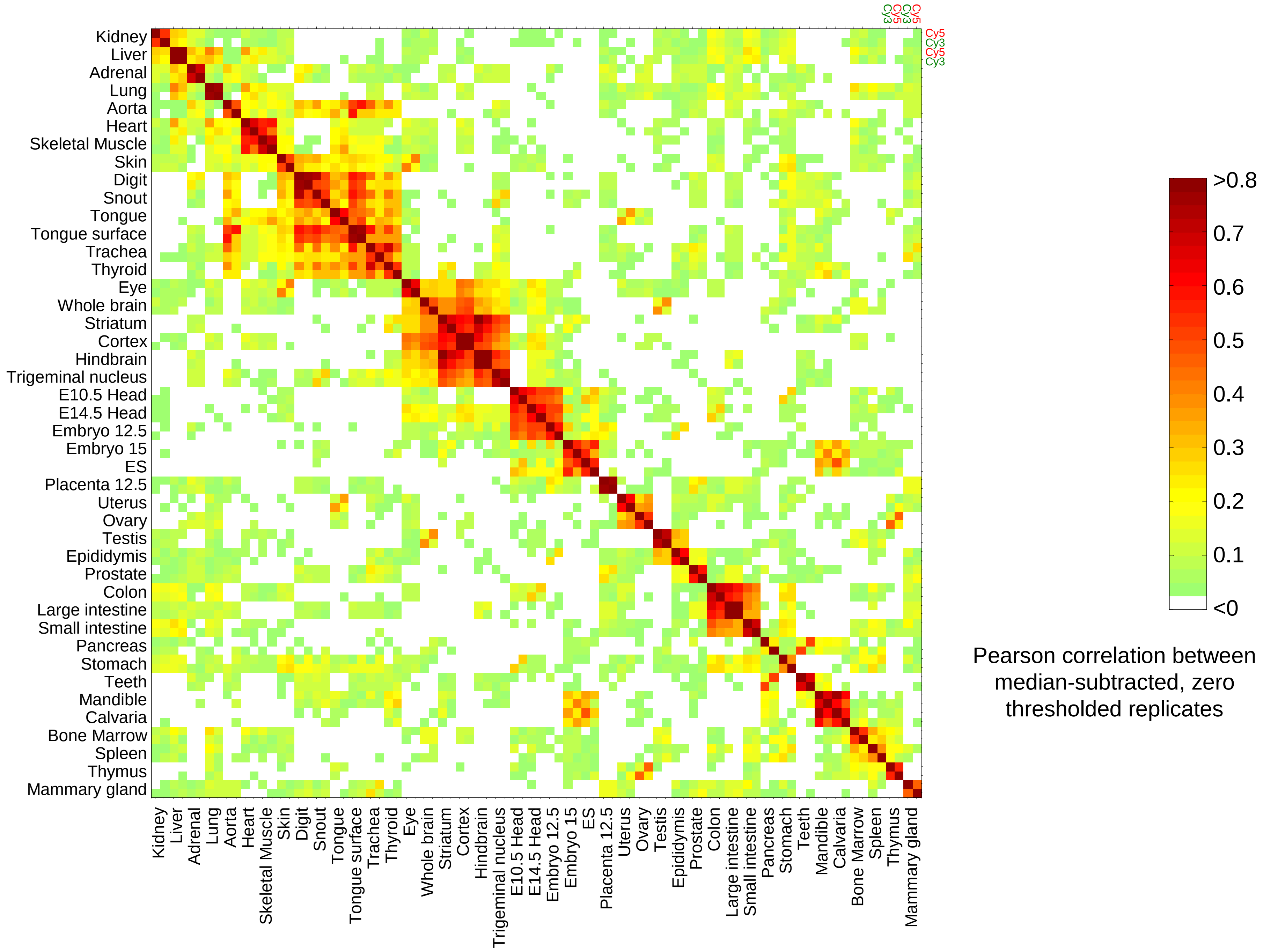
Cy3
Cy5
Cy3
Cy5
Cy5
Cy3
Cy5
Cy3
Kidney
Liver
Adrenal
Lung
Aorta
Heart
Skeletal Muscle
Skin
Digit
Snout
Tongue
Tongue surface
Trachea
Thyroid
Eye
Whole brain
Striatum
Cortex
Hindbrain
Trigeminal nucleus
E10.5 Head
E14.5 Head
Embryo 12.5
Embryo 15
ES
Placenta 12.5
Uterus
Ovary
Testis
Epididymis
Prostate
Colon
Large intestine
Small intestine
Pancreas
Stomach
Teeth
Mandible
Calvaria
Bone Marrow
Spleen
Thymus
Mammary gland
>0.8
0.7
0.6
0.5
0.4
0.3
0.2
0.1
<0
Pearson correlation between median-subtracted, zero thresholded replicates

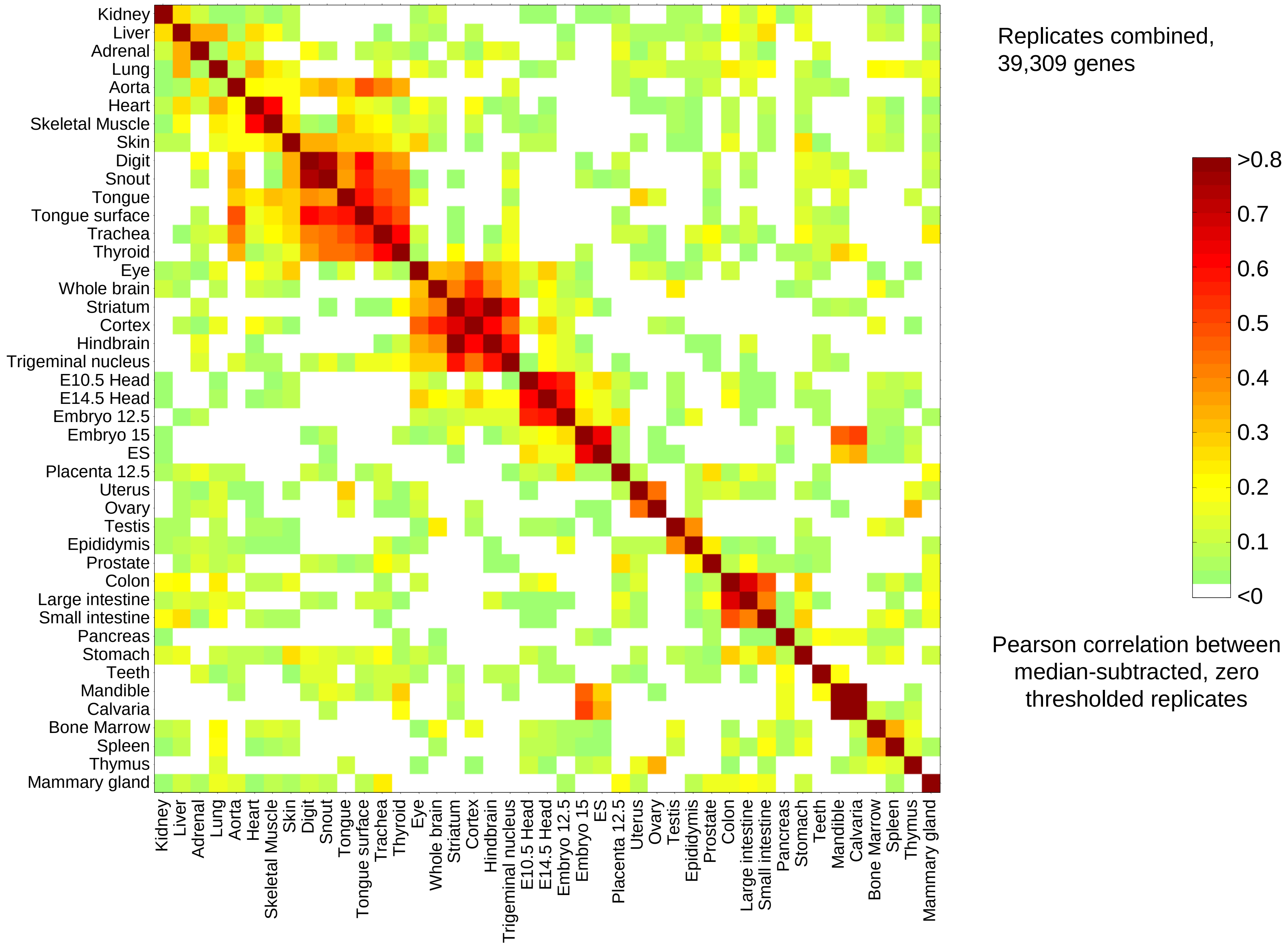
Kidney
Liver
Adrenal
Lung
Aorta
Heart
Skeletal Muscle
Skin
Digit
Snout
Tongue
Tongue surface
Trachea
Thyroid
Eye
Whole brain
Striatum
Cortex
Hindbrain
Trigeminal nucleus
E10.5 Head
E14.5 Head
Embryo 12.5
Embryo 15
ES
Placenta 12.5
Uterus
Ovary
Testis
Epididymis
Prostate
Colon
Large intestine
Small intestine
Pancreas
Stomach
Teeth
Mandible
Calvaria
Bone Marrow
Spleen
Thymus
Mammary gland
Replicates combined, 39,309 genes
>0.8
0.7
0.6
0.5
0.4
0.3
0.2
0.1
<0
Pearson correlation between median-subtracted, zero thresholded replicates

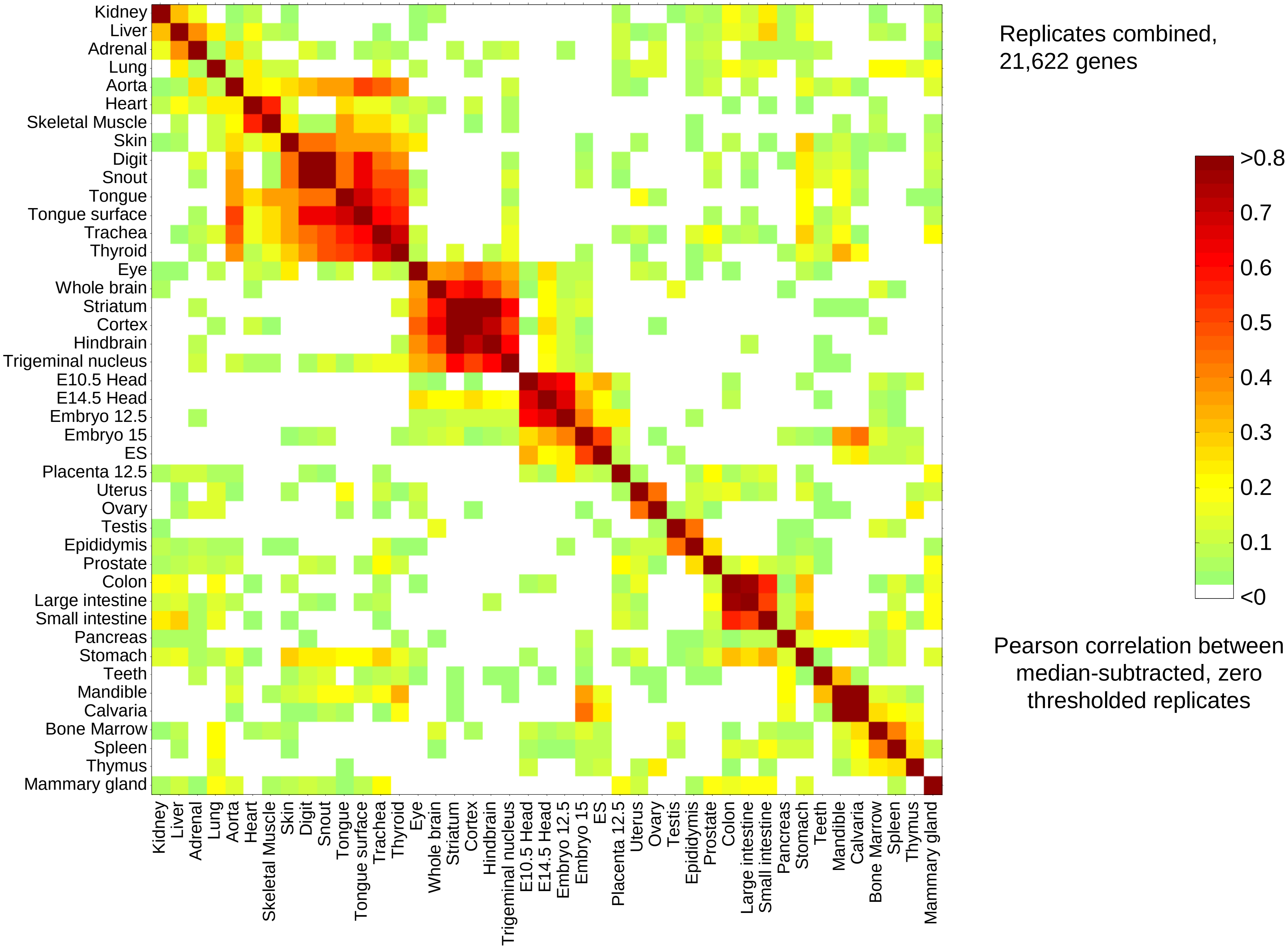
Replicates combined,
21,622 genes
Kidney
Liver
Adrenal
Lung
Aorta
Heart
Skeletal Muscle
Skin
Digit
Snout
Tongue
Tongue surface
Trachea
Thyroid
Eye
Whole brain
Striatum
Cortex
Hindbrain
Trigeminal nucleus
E10.5 Head
E14.5 Head
Embryo 12.5
Embryo 15
ES
Placenta 12.5
Uterus
Ovary
Testis
Epididymis
Prostate
Colon
Large intestine
Small intestine
Pancreas
Stomach
Teeth
Mandible
Calvaria
Bone Marrow
Spleen
Thymus
Mammary gland
>0.8
0.7
0.6
0.5
0.4
0.3
0.2
0.1
<0
Pearson correlation between
median-subtracted, zero
thresholded replicates
