# Supplementary material for: The functional landscape of mouse gene expression
Source: J Biol. 2004 Dec 6;3(5):21. doi: 10.1186/jbiol16 (PMC549719; doi:10.1186/jbiol16)
Supplement: Additional data file 37 — Tech report on spatial detrending [file jbiol16-s37.pdf]

Spatial Bias Removal in Microarray Images

Ofer Shai, Quaid D. Morris, and Brendan J. Frey

October 22, 2003

1 Introduction

Gene expression arrays are revolutionizing the field of molecular biology. The expression levels of thousands of genes can be measured in a single experiment. Already this technology has lead to new genetic disease identification and treatment [2], new cancer treatments [1][3], and a significant number of new findings in molecular biology.

Each microarray, a type of gene expression array, consists of thousands of cDNA probes settled in a grid on an immobile substrate. The amount of mRNA bound to the probes would generally reflect the amount of mRNA transcribed in the sample, which in turn would provide information about the gene expression levels and protein production in the cell. A phosphorous version of the mRNA extracted from the tissue is bound to the probes on the microarray. Laser scanners then excite the dye, causing it to fluorescence and producing an image of the probes.

A major obstacle in analyzing microarray data, and the focus of this paper, is the large amount of noise introduced to the gene expression measurements. This noise stems from multiple sources, such as background leakage, cross-hybridization, uneven sample washing, and scanner biasing.

Here, we introduce a spatial bias removal strategy involving a high pass Gaussian filter. The filter is applied using a two dimensional Fourier transform on the image for computational efficiency. The filtering is performed on the log of the measured intensity values, as there is strong evidence to suggest that the bias is in the form of a multiplicative factor.

2 The spatial bias model

An underlying assumption in our spatial bias removal is that the amount of mRNA bound to one probe of the microarray is independent of the probes around it. Since the probes are generally placed on the microarray in a random permutation, and not corresponding to their corresponding genes' placement in the genome sequence, this assumption is valid whether the microarray was designed to detect complete genes or specific exons in genes. Therefore, visually, the intensity measurements should be independent, and the image obtained should look like random noise.

An inclusive model of all the factors that contribute to the intensity measurement at a certain probe can be written as:

$$\text{observed}_i = \text{true}_i \times \text{bias}_i + \text{noise}_i$$

where we have termed the multiplicative noise as bias, to be distinguished from the additive noise.

The bias is often found as a spatial gradient across the microarray slide, resulting from the scanner, while the noise can generally be attributed to cross-hybridization and other biological sources of error.

2.1 Scanner induced spatial bias

An effective method for observing the effect of the scanner, is to scan the microarray slide twice, but rotate it between scanning by 180°. The rotated image can be restored in software, and the two images compared. Figure 1 shows the result of just such an experiment. We refer to the first scan of the slide as the "original" data, and the second scan, once it has been restored, as the "rotated" data.

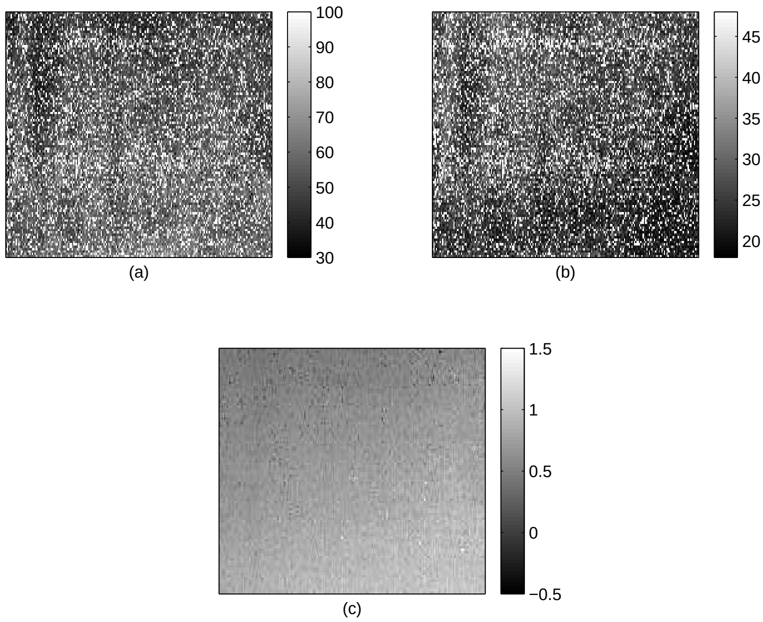

Figure 1: Original and Rotated image. The location of each pixel corresponds to its location on the microarray slide, and the shade corresponds to intensity (in (a) and (b)) or log-difference (in (c)). (a) shows the original image as scanned from the slide , (b) shows the image obtained from the same slide, but scanned rotated 180°. It is easy to see the spatial gradient from the upper left to the lower right corners. (c) shows the log of the ratios of the intensities of the images. Here, the gradient is quite evident - the values should be identical but for scanning errors.

3 Fourier Transform

3.1 2-D Fourier Transform

Fourier transform is a well studied tool in signal processing. The transform allows any signal (or function) to be uniquely transformed into a different domain in a reversible manner. Fourier transform changes a continuous signal from the time domain to the frequency domain. In our case, the time domain signal is an image, and so we must take the Fourier transform as follows:

$$F(u,v) = \int_{-\infty}^{+\infty} \int_{-\infty}^{+\infty} f(x,y) e^{-j(ux+vy)} dxdy \tag{1}$$

and similarly, the inverse Fourier transform is

$$f(x,y) = \frac{1}{2\pi} \int_{-\infty}^{+\infty} \int_{-\infty}^{+\infty} F(u,v) e^{j(ux+vy)} dudv \tag{2}$$

Certain signal processing techniques are easier to perform computationally in the frequency domain. In particular, filtering is quite simple to perform on the transformed signal. The advantage is obtained due to the fact that the convolution of two time domain signals is equivalent to multiplication of their Fourier transforms.

The formulas presented in equations 1 and 2 are valid for continuous signals. However, we are to perform the analysis on a computer where signals can only be represented by discrete sampling. The discrete time Fourier transform (DTFT) for two dimensions is given by:

$$F[n_1,n_2] = \sum_{k_1=0}^{N_1-1} \sum_{k_2=0}^{N_2-1} f[k_1,k_2] e^{-j(\Omega_1 n_1 k_1 + \Omega_2 n_2 k_2} \qquad \begin{array}{l} n_1 = 0,1,2,\ldots,N_1-1 \\ n_2 = 0,1,2,\ldots,N_2-1 \end{array} \tag{3}$$

and

$$f[k_1, k_2] = \frac{1}{N_1 N_2} \sum_{n_1=0}^{N_1-1} \sum_{n_2=0}^{N_2-1} F[n_1, n_2] e^{j(\Omega_1 n_1 k_1 + \Omega_2 n_2 k_2)} \qquad k_1 = 0, 1, 2, \ldots, N_1 - 1$$

$$k_2 = 0, 1, 2, \ldots, N_2 - 1 \tag{4}$$

where N_1 and N_2 are the number of samples (pixels) in the rows and columns of the image, and $\Omega_1 = 2\pi/N_1$, $\Omega_2 = 2\pi/N_2$.

3.2 The effect of discretization

In 1-dimensional Fourier transform, a discrete time signal generates a periodic Fourier transform, and similarly, a discrete frequency signal indicates a periodic time signal. The effect of using a computer to analyze continuous, time limited signals, is to implicitly make them periodic in both the time and frequency domains. The assumption, then (through the mathematics of Fourier transforms) is that the sample we are concerned with is only the first repetition of a continuous time signal.

similarly, when analyzing 2D images, the Fourier transform obtained is that of a periodic image obtained by tiling the image in a non-overlapping grid. This becomes extremely important when applying the filter. In figure 2(b) we can see that after applying a low pass Gaussian filter as discussed in section 3.3) to the image in figure 2(a), the black boxes have been blurred into the white background. The boxes around the edge of the image have "leaked" to the other side of the image. From the discussion above, the reason for that should be obvious - the bottom and right edges of the image "wrap around" to the top and left edges respectively due to the inherent periodicity of the image. To adjust for the periodicity of the image, we flip and tile the image, as shown in

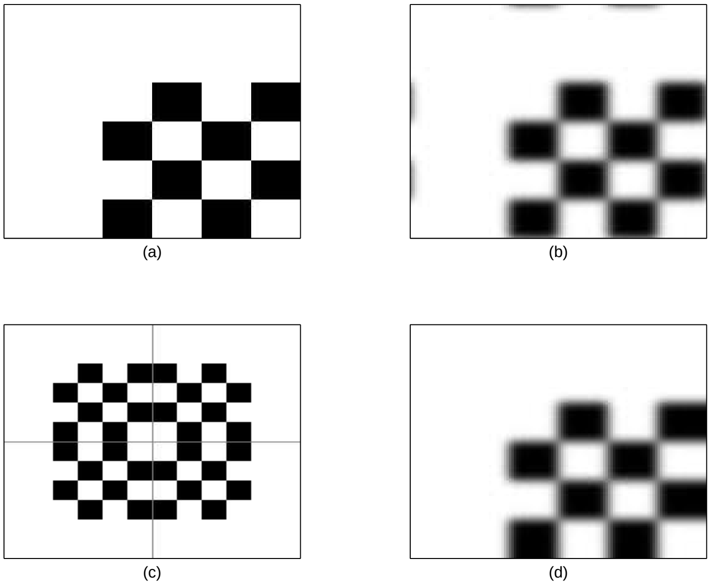

Figure 2: The effect of a low pass filter on an image. (a) shows the original image, (b) shows the image after a low pass filter was applied to the Fourier transform. (c) shows the image tiled. Finally, (d) shows the result of the filtering on the tiled image, cropped to the area of the original image.

figure 2(c),and the post filtering image does not display any ”leakage” at the edges, as is seen in 2(d). The tiled image is still considered as periodic, but each edge is now facing itself.

3.3 Gaussian filtering in frequency domain

The high pass filtering was performed using a Gaussian filter. Experiments were performed using Gaussian, Hamming, and rectangular filters. Gaussian had the most desirable effect. As mentioned in section 3.1, multiplication in the frequency domain is equivalent to convolution in time domain. The Gaussian high pass filter becomes intuitively clear - a blurred version of the image, produced with a Gaussian blur tool, is subtracted from the original.

Previously, a blurred version of the image produced by a 3×3 median window was found to perform spatial bias removal with some degree of improvement [4]. The important differences in the current work are that

a. The filtering is performed on a Fourier transformed image, making it much more computationally efficient

b. The Gaussian window is not clipped at a set amount. Instead, the Gaussian extends to the entire image. Of course, the width of the relevant part near the center of the Gaussian can be adjusted.

c. The filtering is performed on the log of the image, thereby removing the *multimplicative* bias.

4 Results

Once again, we will focus on the rotated slide data (see section 2.1 for details), as we can use it to gauge the effectiveness of the spatial bias removal.

To measure our confidence in the filter, we measured the mean squared error (MSE) in the log of the ratios of the two original and rotated data before and after filtering. The pre-filtering MSE is 6.21×10^{-3}. Using the Gaussian filter discussed in section 3.3, with a standard deviation parameter being 0.3 of the width and length of the image, the post-filtering MSE achieved was 0.837×10^{-3}, a 7.5-fold improvement. The scatter plot for before and after filtering is shown in figure 4. The plots in figure 4 do not pass through the origin, indicating that the two data sets are on different scales. This is most likely due to difference in laser intensities while scanning and can be corrected using inter-slide normalization.

One may make the argument that the two slides have a higher agreement because they are much more "alike" after filtering. For example, a constant output filter would produce a MSE of 0. To allow for that possibility, we mea-

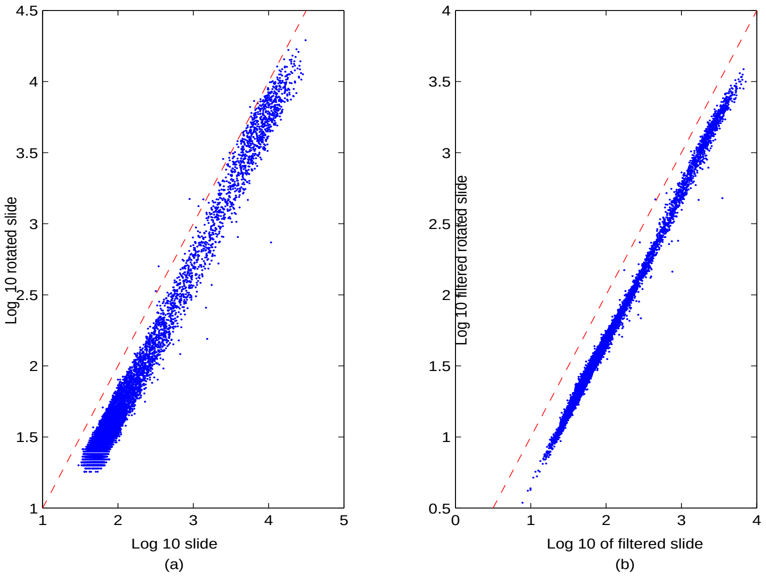

Figure 3: Scatter plots of (a) log intensity of original data Vs. rotated data, pre-filtering, and (b) log intensity of original data Vs. rotated data, post-filtering. The dashed red line is where ideally the data should lie. In both plots, the data is below and parallel to the straight line through the origin, indicating a negative offset in log domain (a slope ¡ 1 in intensity). The width of the scatter plot is an indication of the MSE, and is much thinner in (b).

sured the MSE of the original data with a randomly permutation of the rotated data. The original MSE was calculated to be 0.488, while the post-filtering MSE was calculated to be 0.286, a 1.7-fold ”improvement”. We must conclude that although the 7.5-fold improvement reported above is somewhat optimistic, it is significant.

The spatial bias removal was also carried out on mouse genes experiments performed at Dr. Timothy Hughes’ Lab at the Best Institute, Toronto. There, we do not have a clear method of evaluating the quality of the results. We did, however, visually examine the results, as well as compared values from different experiments involving the same biological samples. The results were comparable with those described above.

5 Summary

We developed a new new technique for removing spatial bias. In particular, bias introduced by the scanning equipment, or similar multiplicative, low-frequency bias, can be removed quite efficiently. We presented detailed results for a single slide that was scanned twice, at 180° rotations, and showed a 7.5-fold increase in signal-to-noise ration (SNR).

The key result pertaining to this process is the observation that there is a large amount of low frequency multiplicative bias in the micro array data.

References

[1] M. Basik, S. Mousses, and J. Trent. Integration of genomic technologies for accelerated cancer drug development. *Biotechniques*, 35(3):580–2, 584, 586, 2003.

[2] G. Firneisz, I. Zehavi, C. Vermes, A. Hanyecz, J. A. Frieman, and T. T. Glant. Identification and quantification of disease-related gene clusters. *Biotechniques*, 19(14):1781–6, 2003.

[3] M. A. Shipp, K. N. Ross, P. Tamayo, A. P. Weng, J. L. Kutok, R. C. Aguiar, M. Gaasenbeek, M. Angelo, M. Reich, G. S. Pinkus, T. S. Ray, M. A. Koval, K. W. Last, A. Norton, T.A. Lister, J. Mesirov, D. S. Neuberg, E. S. Lander, J. C. Aster, and T. R. Golub. Diffuse large b-cell lymphoma outcome prediction by gene-expression profiling and supervised machine learning. *Nature Medicine*, 8(1):68–74, 2002.

[4] D. L. Wilson, M. J. Buckley, C. A. Helliwell, and I. W. Wilson. New normalization methods for cdna microarray data. *Bioinfomatics*, 19:1325–1332, 2003.
